# The novel Aryl hydrocarbon receptor inhibitor biseugenol inhibits gastric tumor growth and peritoneal dissemination

**DOI:** 10.18632/oncotarget.2307

**Published:** 2014-08-04

**Authors:** De-Wei Lai, Shing-Hwa Liu, Anna Isabella Karlsson, Wen-Jane Lee, Keh-Bin Wang, Yi-Ching Chen, Chin-Chang Shen, Sheng-Mao Wu, Chia-Yu Liu, Hsing-Ru Tien, Yen-Chun Peng, Yee-Jee Jan, Te-Hsin Chao, Keng-Hsin Lan, Jack L. Arbiser, Meei-Ling Sheu

**Affiliations:** ^1^ Institute of Biomedical Sciences, National Chung Hsing University, Taichung, Taiwan; ^2^ Institute of Toxicology, College of Medicine, National Taiwan University, Taipei, Taiwan; ^3^ Department of Dermatology, Emory University School of Medicine, Winship Cancer Institute, Atlanta Veterans Administration Health Center, Atlanta, Georgia, USA; ^4^ Department of Medical Research, Taichung Veterans General Hospital, Taichung, Taiwan; ^5^ Department of Nuclear Medicine, Kuang Tien General Hospital, Taichung, Taiwan; ^6^ Institute of Nuclear Energy Research, Atomic Energy Council, Longtan, Taoyua, Taiwan; ^7^ Division of Gastroenterology, Department of Internal Medicine, Taichung Veterans General Hospital, Taichung, Taiwan; ^8^ Department of Pathology and Laboratory Medicine, Taichung Veterans General Hospital, Taichung, Taiwan; ^9^ Division of Colorectal Surgery, Department of Surgery, Taichung Veterans General Hospital, Taichung, Taiwan; ^10^ Department and Institute of Pharmacology, National Yang-Ming University, Taipei, Taiwan; ^11^ Division of Gastroenterology, Department of Medicine, Taipei Veterans General Hospital, Taipei, Taiwan; ^12^ Rong Hsing Research Center for Translational Medicine, National Chung Hsing University, Taichung, Taiwan

**Keywords:** ER stress, Calpain-10, AhR, Snail, EMT

## Abstract

Biseugenol (Eug) is known to antiproliferative of cancer cells; however, to date, the antiperitoneal dissemination effects have not been studied in any mouse cancer model. In this study, Aryl hydrocarbon receptor (AhR) expression was associated with lymph node and distant metastasis in patients with gastric cancer and was correlated with clinicolpathological pattern. We evaluated the antiperitoneal dissemination potential of knockdown AhR and Biseugenol in cancer mouse model and assessed mesenchymal characteristics. Our results demonstrate that tumor growth, peritoneal dissemination and peritoneum or organ metastasis implanted MKN45 cells were significantly decreased in shAhR and Biseugenol-treated mice and that endoplasmic reticulum (ER) stress was caused. Biseugenol-exposure tumors showed acquired epithelial features such as phosphorylation of E-cadherin, cytokeratin-18 and loss mesenchymal signature Snail, but not vimentin regulation. Snail expression, through AhR activation, is an epithelial-to-mesenchymal transition (EMT) determinant. Moreover, Biseugenol enhanced Calpain-10 (Calp-10) and AhR interaction resulted in Snail downregulation. The effect of shCalpain-10 in cancer cells was associated with inactivation of AhR/Snail promoter binding activity. Inhibition of Calpain-10 in gastric cancer cells by short hairpin RNA or pharmacological inhibitor was found to effectively reduced growth ability and vessel density *in vivo*. Importantly, knockdown of AhR completed abrogated peritoneal dissemination. Herein, Biseugenol targeting ER stress provokes Calpain-10 activity, sequentially induces reversal of EMT and apoptosis via AhR may involve the paralleling processes. Taken together, these data suggest that Calpain-10 activation and AhR inhibition by Biseugenol impedes both gastric tumor growth and peritoneal dissemination by inducing ER stress and inhibiting EMT.

## INTRODUCTION

Epithelial-to-mesenchymal transition (EMT) is a pivotal mechanism in embryonic development and peritoneal dissemination [1;2]. Recently studies have shown that endoplasmic reticulum (ER) stress can directly exert an effect on cancer cells themselves, or on endothelial cells, reducing tumor growth, metastasis, and inducing cell cycle arrest, senescence, apoptosis [2-6]. Peritoneal dissemination is the most common cause of metastasis from malignancies, such as gastric, colon, ovarian, pancreatic cancer, and patients with this condition are generally refractory to conventional therapeutic approaches [7-10]. Yet, no effective therapy has been established so far to alleviate this devastating and often fatal end-stage condition. Therefore, there is an urgent need to seek for novel therapy for suppresses cancer peritoneal dissemination and mitigate suffering advanced cancer patients.

Aryl hydrocarbon receptor (AhR) functions as a ligand-activated basic helix-loop-helix transcription factor regulating transcription encoding xenobiotic metabolizing enzymes, which has been shown constitutively active AhR induces stomach tumors [11;12]. Importantly, previous studies have demonstrated that AhR participate in tumor initiation, promotion and progression [13]. AhR exposure to 7,12-dimethylbenz[a]anthracene (DMBA), has recently been shown to activate transcription of Slug, another repressor of E-cadherin gene transcription, suggesting a signaling mechanism may contribute to EMT in mammary epithelial cell and MDCK cells [13-17]. Overexpressing AhR in human mammary epithelial cells (HMEC) exhibited enhanced motility, migration and invasion [18]. Paradoxically, there is substantial evidence that it may act as a converse role. Staršíchová A et al. have shown that tumorigenesis inducer transforming growth factor β-1 (TGF-β1), suppresses the AhR-mediated gene expression through multiple mechanisms, involving inhibition of AhR expression and down-regulation of nuclear AhR, via a SMAD4-dependent pathway in prostate epithelial cells [19]. Rico-Leo EM et al. demonstrated AhR(−/−) keratinocytes and sh-AhR NMuMG cells derived from normal mouse mammary epithelial cells had increased migration, reduced levels of epithelial markers, and increased expression of mesenchymal markers [20]. In addition, basal or TGFβ-induced AhR down-modulation could be relevant in the acquisition of a motile EMT phenotype in both normal and transformed epithelial cells [20]. Recently, our reports have shown that inducing ER stress dampening peritoneal dissemination and inhibiting of angiogenesis [2;5;21]. However, the role of AhR on EMT and cellular and molecular mechanisms of the development, progression, and peritoneal dissemination in gastric cancer still remain to be clarified.

Biseugenol (4-allyl-2-methoxyphenol; Eug), one of phenolic phytochemicals, is a biologically active phenolic component of *Syzigium aromaticum* (cloves), which has been shown to be a potential anticancer agent in multiple facets of signal transduction and possess various biological properties such as antiviral, antioxidant, anti-inflammatory, etc [22;23]. World Health Organization (WHO) Food and Agriculture Organization (FAO) have admitted an acceptable daily intake of Biseugenol of 2.5 mg/kg body weight for humans [24]. Biseugenol has been considered non-carcinogenic and non-mutagenic and announced as safe by the U.S. Food and Drug Administration (FDA). Ghosh R et al. have shown that Biseugenol causes melanoma growth suppression through inhibition of E2F1 transcriptional activity [25]. Nangia-Makker P and colleagues demonstrated that inhibits tumor growth and angiogenesis in MDA-MB-231 cells [26]. Inhibitory effects of Biseugenol on the activity and expression of MMP-9 activity related to metastasis has also been found by Nam H [27]. In addition, Biseugenol acts as a potent inhibitor of NF-κB, prevention of lipopolysaccharide-stimulated macrophages activation and inflammatory cytokine expression [28]. We previous reported that activating ER stress thwarts gastric tumor growth, peritoneal dissemination through inducing apoptosis and reversal EMT process [2;5;21;29]. The unfolded protein response (UPR) is a cellular stress response related to the endoplasmic reticulum stress, was shown to require in *nu/nu* mice microvasculature for treating breast tumor with ER stress- activator tunicamycin by Aditi Banerjee et al. demonstrated [3]. However, the effects of Biseugenol on ER stress correlated tumor growth and peritoneal dissemination are still unclear. Herein, we hypothesize that Biseugenol inhibits the EMT progression of gastric cancer cells through a Calpain-10- interaction with AhR and regulated Snail pathway. Taken together, these findings suggest that the therapeutic activation of Calpain-10 by Biseugenol-treated and further interaction with AhR suppresses both gastric tumor growth and peritoneal dissemination by inducing ER.

## RESULTS

### Aryl hydrocarbon receptor (AhR) is upregulated in gastric cancer tissues and gastric cancer cell lines

**Figure 1:**
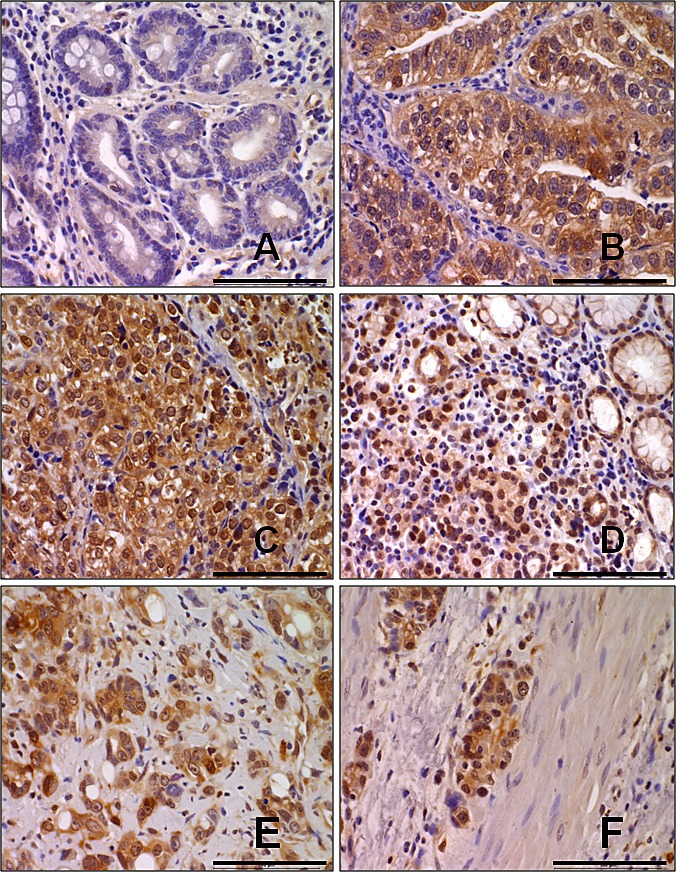
AhR is up-regulated in gastric cancer Representative immunostaining of AhR expressions in human normal gastric mucosa (A), moderately differentiated intestinal type adenocarcinoma (B), poorly differentiated intestinal type adenocarcinoma (C), diffused type adenocarcinoma in mucosa (D), diffuse type adenocarcinoma in serosa (E), and diffuse type adenocarcinoma in muscularis propria (F) were shown. Scale bar= 10 μm.

To investigate a possible role for AhR in gastric cancer progression, we performed immunohistochemical analysis of 40 patient's human gastric cancer specimens and demonstrated increase in AhR expression, as compared with benign tissue adjacent to the tumor (Figure 1A). After surveying benign tissue, typical moderately differentiated adenocarcinoma (Figure 1B) and poorly differentiated signet-ring cell carcinoma (Figure 1C) make up the majority of tumors shown in gastric cancer specimen. In the diffused-type gastric cancer tissues (Figure.1D), adenocarcinoma with omentum metastasis (Figure 1E), adenocarcinoma with lymph node and distant metastasis (Figure 1F). The percentage of positive tumor cells and the staining intensity for each sample were recorded. The clinicopathological characteristics of the gastric cancer patients are summarized in Table 1. The high expression rate of the AhR was 67.5% (27/40) in gastric cancer case and low expression rate 32.5% (13/40) in neoplastic tissues. A significant statistical difference was found between the two groups. The level of AhR expression closely correlated with increased clinical stage as well as with lymph node and distant metastasis of tumor-node-metastasis (TNM) classification, respectively. Furthermore, protein level AhR expression different in human stomach cancer epithelial cell line (AGS, MKN45, N-87, SCM-1), human colon cancer epithelial cell line (HCT116) and normal cells (AMJ2, MMC, SVECs, HUVECs). Highly metastasis cells MKN45 and HCT116 expressed higher AhR, and normal cells expressed lower AhR (Supplementary Fig.1).

**Table 1:** Correlation between AhR expression and clinicopathological characteristics of gastric cancer

		AhR expression				AhR expression	
Characteteristics	Case (N=40)	Low (N=13)	High (N=27)	*P*-value	Case %	Low 32.50%	High 67.50%
Gender				0.502			
Male	26	10	16		65	25	40
Female	14	3	11		35	7.5	27.5
Age (years)				0.404			
≤60	28	8	20		70	20	50
>60	12	5	7		30	12.5	17.5
Differentiation				0.249			
well	8	1	7		20	2.5	17.5
Moderately	14	5	9		35	12.5	22.5
Poorly	18	7	11		45	17.5	27. 5
lauren classification				0.529			
Intestinal	18	5	13		45	12.5	32.5
Diffuse	22	8	14		55	20	35
T classification				0.035			
T1	3	1	2		7.5	2.5	5
T2	5	2	3		12.5	5	7.5
T3	22	7	15		55	17.5	37.5
T4	10	3	7		25	7.5	17.5
N classification				0.024			
N0	10	3	7		25	7.5	17.5
N1	14	5	9		35	12.5	22.5
N2	12	4	8		30	10	20
N3	4	1	3		10	2.5	7.5
Distant metastasis				0.032			
Negative	26	11	15		65	27.5	37.5
Positive	14	2	12		35	5	30
TNM stage				0.036			
I	4	1	3		10	2.5	7.5
II	10	4	6		25	10	15
III	12	3	9		30	7.5	22.5
IV	14	5	9		35	12.5	22.5

### Biseugenol suppresses gastric tumor growth, peritoneal dissemination and organ metastasis involving in ER stress

**Figure 2:**
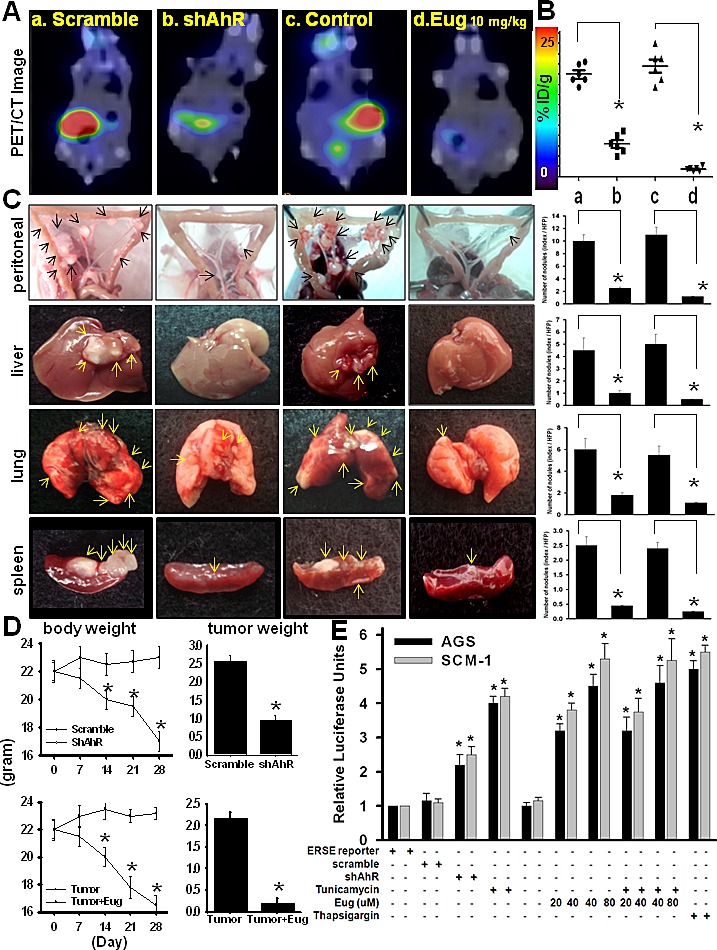
Effects of Biseugenol on gastric tumor growth, peritoneal dissemination, organ metastasis, and ER stress activity (A) [^18^F]-FDG-PET presented as a surrogate measure of therapeutic utility. Tumors in nude mice were established for 7 days after the intraperitoneal inoculation of MKN45 gastric cancer cells (5×10^5^/mice). Mice were then injected intraperitoneally with Biseugenol (Eug, 10 mg/kg/twice per week). Thirty days after Biseugenol treatment, the images of [^18^F]-FDG-PET-PET/CT in mice were captured, and then mice were sacrificed for macroscopic examination of the distribution of disseminated metastasis. Transfections of siRNA-AhR and scramble into MKN45 were performed by Lipofectin. Representative images of [^18^F]-FDG-PET-PET/CT in mice inoculated with MKN45 gastric cancer cells with or without Biseugenol treatment were shown. (B) Quantifications of estimated radioactivity intensity. Note different maximum values on top and bottom PET color scale bars. The maximum intensity projection of typical representative nude mice (a, scramble, b. siRNA-AhR, c, control, d, Biseugenol treatment was shown. (C) A representative images showing peritoneal dissemination of control and Biseugenol-treated animals were presented. The occurrence of distant metastases was found in the organs of liver, lung, and spleen as indicated. In contrast, peritoneal metastasis was observed sporadically in Biseugenol-treated mice. Quantifications of number of nodules (index/HFP) were estimated by H&E staining (*Right panel*). (D) Body weight (Left panel) and tumor weight (right panel) per mice were assessed as indicated. Data are presented as mean±SEM (n =12). (E) ER stress activity by ERSE reporter detection. ERSE reporter is designed to measure activity of endoplasmic reticulum (ER) stress signaling. AGS cells were transfected with ERSE reporter, negative control and positive control. After 20 hours of transfection, cells were treated with either 0.1 μg/ml of tunicamycin or 10 nM thapsigargin for 16 hours. Dual Luciferase assay was performed 36 hours after transfection, and promoter activity values are expressed as arbitrary units using a Renilla reporter for internal normalization. Experiments were done in triplicates, and the standard deviation is indicated. Data shown are representative of at least three independent experiments.

In this study, we examined whether Biseugenol could suppress gastric tumor growth, peritoneal dissemination of gastric cancer along with major organ metastasis. Actually, Monoeugenol treatment did not induce the cell morphological changes and other functional effect (Supplementary Figure 2). We silenced AhR as a positive control [12]. To evaluate the effect of Biseugenol *in vivo* activity in terms of primary tumor growth*,* peritoneal dissemination and distant organ metastasis of, mice were implanted with MKN45 cells and SCM-1 (data not shown) into the abdominal cavity of nude mice. We found that Biseugenol-treated tumor (10 mg/kg/twice/weekly) decreased primary tumor growth, peritoneal extension, and liver, lung and spleen macro-metastasis by 85-90 % compared with that of the control tumor group by PET/CT imaging and quantification the tumor burden (Figure 2A-2B). Importantly, Biseugenol treatment inhibited intestinal mesentery nodules and various organs effects are superior than silencing AhR mice (Figure 2C). Quantification of Biseugenol-inhibited peritoneal metastasis of gastric tumor cells is shown in right panel. Biseugenol had no toxic effects and in fact induced modest weight gains. Quantification of body weight (left panel) and tumor weights (right panel) is shown in (Figure 2D).

A recent novel finding is that induction ER stress can inhibit peritoneal dissemination [2-6]. Because of the emerging role of ER stress induction as a therapeutic modality, we investigated whether Biseugenol induced ER stress using the Cignal ERSE Reporter Assay. As shown in Figure 2E, exposure of gastric cancer cells to Biseugenol, led to a 3-5.2 fold induction, as compared with control cells, proving a critical clue that Biseugenol induces ER stress in gastric cancer. Similar results were observed with known inducers of ER stress tunicamycin and thapsigargin. Combination Biseugenol and tunicamycin did not have markedly synergistic effect in ERSE Reporter Assay and viability effect (Figure 2E and Supplementary Figure 3A). In addition, the activity was observed in knockdown AhR compared with control groups (Figure 2E).

### Calpain-10 regulates AhR expression by Biseugenol treatment

**Figure 3:**
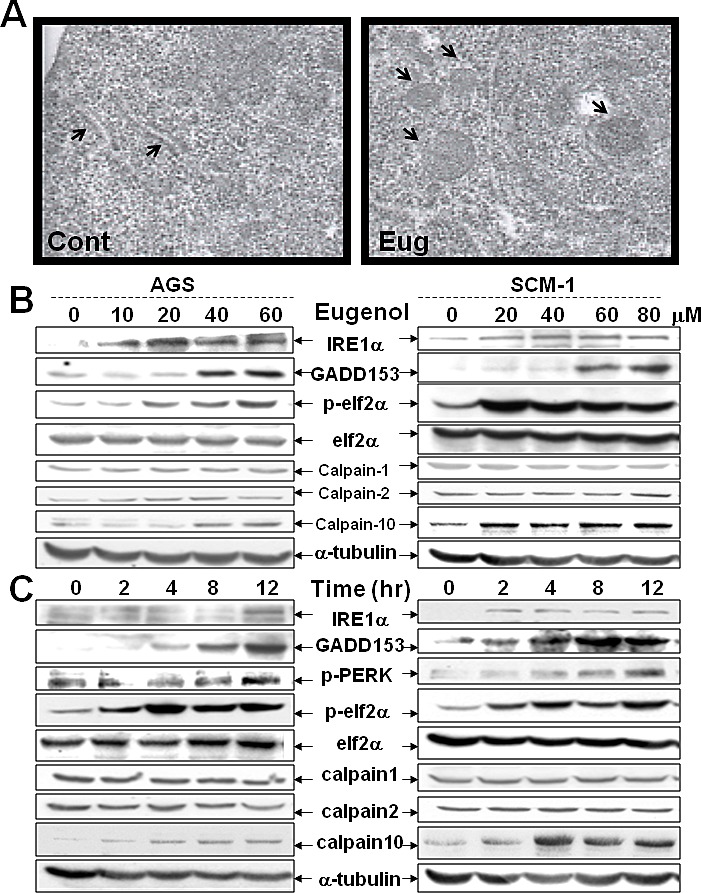
Biseugenol induces ER stress in gastric cancer cells (A) Gastric cancer cells were treated with or without Biseugenol (Eug, 80 μM) for 20 h. MKN45 cells were collected and visualized by electron microscopy as described in “Materials and methods”. The images shown are representative of two independent experiments. a, Control cells; b, Biseugenol-treated cells displays ER swelling and ER organelles dilation. Arrows indicate dilated ER. Original magnifications: ×800 K. (B) Treatment with Biseugenol for 24 h in gastric cancer cells induces ER stress-related molecules in a dose-dependent manner. (C) Treatment with Biseugenol in gastric cancer cells induces ER stress-related molecules in a time-dependent manner.

**Figure 4:**
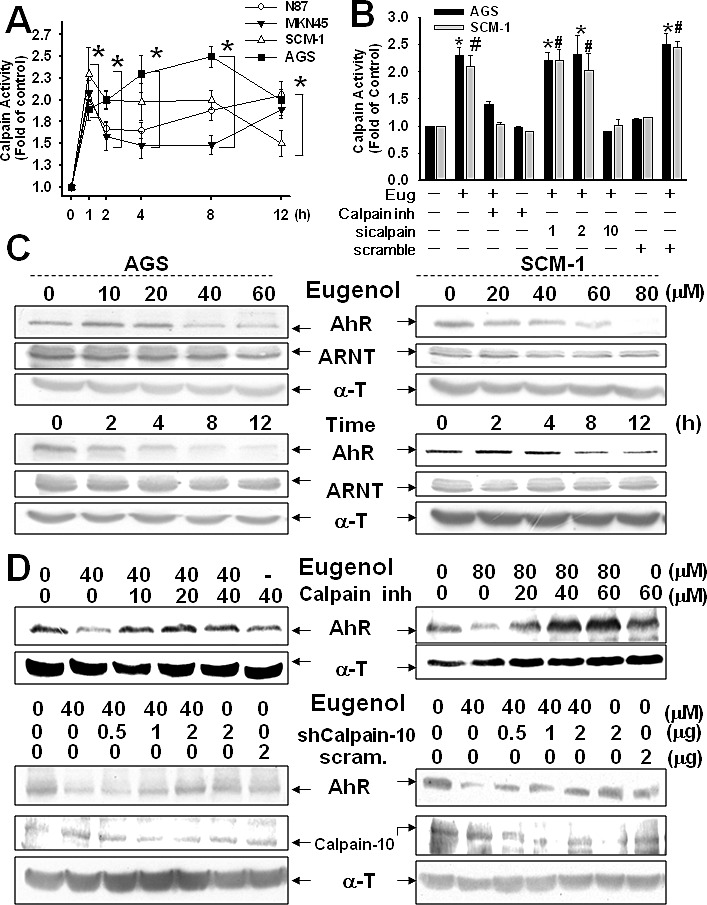
Calpain-10 regulates AhR expression in Biseugenol-treated gastric cancer cells (A). Calpain activity was measured with the fluorescent calpain substrate Suc-LLVY-AMC in N87, AGS, MKN45, and SCM-1 cells. (A) Time course responses to Biseugenol (Eug) (40 μM in N87 and AGS,; 80 μM in MKN45 and SCM-1) treatment. Data are expressed in terms of fold of control conditions. (B) Transfection with shCalpain-10 but not shcalpain-1, shcalpain-2 or scramble reduces the increased calpain activity in AGS cells treated with Eug for 8 h. Calpain inhibitor Z-Leu-Leu-CHO; 25 μM) significantly inhibited Eug-increased calpain activity. Data are presented as mean±SEM (n = 5). (C) AGS and SCM-1 cancer cells were treated with Eug either in a dose- or time-dependent manner, and then AhR and Arnt expressions were evaluated by Western blotting. (D) The expressions of AhR or Calpain-10 in gastric cancer cells (AGS and SCM-1) with or without Biseugenol (AGS, 40 μM or SCM-1 80 μM) treatment for 24 h in the presence or absence of a Calpain inhibitors Z-Leu-Leu-CHO or Calpain-10 siRNA transfection were detected. The results shown are representative of at least four independent experiments.

To more directly assess the effect of Biseugenol on organelle morphology, we utilized TEM proved ER stress, straightforwardly. In Figure 3A, we demonstrated there was ER dilation and swelling in Biseugenol-treated cells, suggestive of ER stress. Next, we investigated the correlation with molecular ER stress marker. We found that Biseugenol induces the canonical ER stress markers IRE1α, GADD153, p-elf2α in dose-dependent manner and time-dependent effect. Noticeably, we observed a significant increase in Calpain-10 expression, but not calpain-1 or calpain-2 expression (Figure 3B-3C). Thus, we evaluated the effect of Biseugenol on calpain activity. As shown in Figure. 4A, Biseugenol increased calpain activity in a time-dependent manner, which started to increase at 30 min, and peaked at 60 min, and then was sustained to 12 h, followed by a decrease at 24 h. Cells remained adherent over the time course, with no loss of viability.

To confirm Calpain-10 specific functions, we further tested whether knockdown specific Calpain-10 activity could compromise the ability of Biseugenol on activation function induction. As shown in Figure. 4B, Biseugenol induced calpain activity in gastric cancer cells, which could be reversed by calpain inhibitor (ALLN). Importantly, using a siRNA to Calpain-10 activity led to a significant abatement of Biseugenol-induced activity in human gastric cancer cells after 1 h treatment. SiRNA-calpain-1 or calpain-2 did not affect Biseugenol-induced activity. Therefore, Biseugenol is specific in its ability to induce ER stress via atypical ER stress-Calpain-10 mechanism.

An important role of the AhR has been reported consistent with gastric tumorigenesis and thus also in the control of growth and proliferation of gastric epithelial cells [12]. We previously demonstrated that in Figure2 that using a siRNA to knockdown AhR led to a markedly inhibits metastasis *in vivo*. Next, we hypothesis that Biseugenol-induces Calpain-10 activity regulates AhR expression, resulting in inhibiting metastasis proceed and peritoneal dissemination. As shown in Figure 4C, Biseugenol specifically decreased AhR in a dose-dependent effect and time-dependent manner, but not ARNT (aryl hydrocarbon receptor nuclear translocator protein), that forms a complex with ligand-bound AhR. Consistent with our hypothesis, pretreatment calpain inhibitor significantly reversed AhR production. Transfection cancer cells with siRNA-Calpain-10, but not scramble siRNA (data not shown), caused reversion of AhR down-regulation (Figure 4D). These results also demonstrated that Biseugenol-mediated Calpain-10 regulated AhR expression.

### Biseugenol enhances Calpain-10 /AhR interaction

**Figure 5:**
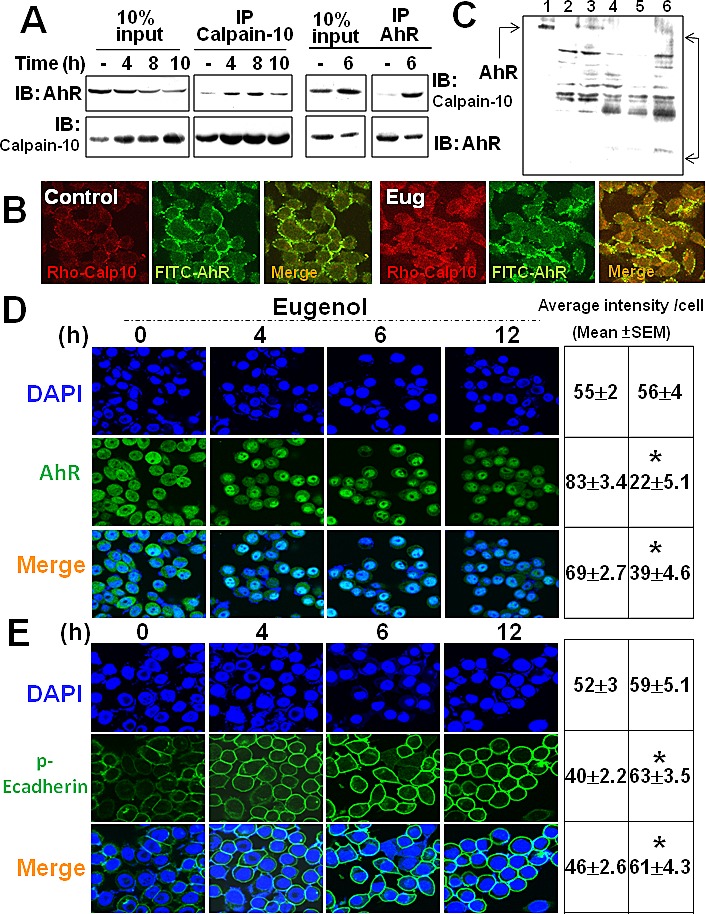
Biseugenol enhances Calpain-10 /AhR interaction and phosphorylation of Ecadherin in gastric cancer cells (A). AGS were treated with or without Biseugenol (Eug, 40 μM) for 4, 8 or 10 h. Calpain-10 was immunoprecipitated by anti-Calpain-10 antibody from cell lysates and the immunoblot was probed with the antibodies for AhR and Calpain-10. The results shown are representative of three independent experiments. (B) Laser confocal microscopy for Calpain-10 and AhR interaction in SCM-1. Cells were exposed to Biseugenol (40 μg/ml) for 8 h and then fixed and incubated with monoclonal antibodies against specific Calpain-10 and AhR. (C) Proteolysis of AhR in the presence of Calpain-10. Purified AhR were digested by the indicated concentrations of recombinant Calpain-10 at 37°C for 4 h in the presence of 0.5 mM CaCl_2_. Samples were analyzed by 8% SDS-PAGE and stained with Coomassie blue. Data shown are from one experiment representative of at least three performed. Lane1, Purified AhR protein; Lane2, recombinant Calpain-10, 0.1 U/ml; Lane3, 0.5 U/ml; Lane4, 1 U/ml; Lane5, 5 U/ml; Lane6, 10 U/ml. (D) Laser confocal microscopy for AhR or (E) phosphorylation of Ecadherin in SCM-1. Cells were exposed to Biseugenol (40 μM) for 4, 6 and 12 h and then fixed and incubated with monoclonal antibodies against specific AhR and p-Ecadherin. Confocal images for AhR and p-Ecadherin were examined and quantified as described in the Materials and methods section. The images are representative of at least five independent experiments. The average intensity per cell is presented as mean ± SEM.

We further examined whether Biseugenol enhances the interaction of Calpain-10 with AhR. As shown in Figure 5A, the interaction of Calpain-10 with AhR determined by immunoprecipitation assay was modest in the control cells, yet their interaction was significantly enhanced and lasted 4-8 h in the presence of Biseugenol. A comparison of 10% input displayed for protein visualization was used as a positive control. In addition, confocal microscopy detection revealed that exposure in 8 h efficiently enhances the co-localization of Calpain-10 with AhR in Biseugenol-treated SCM-1 cells (Figure 5B). In order to determine whether Calpain-10 cleaved AhR, we digested concentrated AhR with recombinant Calpain-10 under various conditions *in vitro* (Figure 5C). Incubation of AhR with recombinant Calpain-10 for 1 h at 30 °C led to the complete digestion of full-length AhR as determined by *in vitro* cleavage assay. The cleaved fragments depended on the presence of recombinant Calpain-10. We also assessed transfected Calpain-10 siRNA knocks down the co-localization of endogenous Calpain-10/AhR in Biseugenol-treated gastric cancer cells (data not shown). These results indicated that Biseugenol enhances Calpain-10/AhR interaction, leading to cleavage of AhR.

### Biseugenol inhibits EMT marker *in vitro* via AhR down-regulation

**Figure 6:**
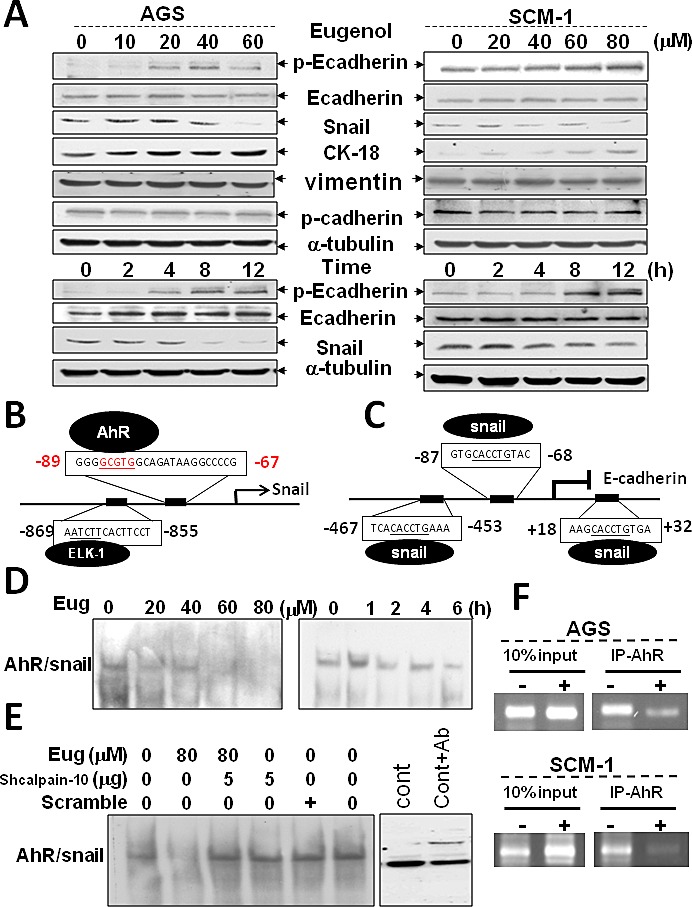
Biseugenol inhibits EMT *in vitro* via AhR down-regulation (A) AGS and SCM-1 cancer cells were treated with Biseugenol (Eug) either in a dose- or time-dependent manner. EMT markers (p-Ecadherin, cytokeratin-18, Snail, vimentin, and p-cadherin) were evaluated by Western blotting. The sequences of Snail and Ecadherin promoter are presented (B-C), indicating the sub-fragments that have been used in this study (arrowheads), and the Snail minimal promoter (underlined). Promoter regulation in the Snail promoter-flanking region (−89~−67) contains the *cis*-acting elements AhR DNA binding site. E-cadherin promoter-flanking region (−87~−68) contains the transcription repression Snail DNA binding site. (D) Cancer cells were treated with or without Biseugenol (40 μg/ml) for 6 h, and then nuclear AhR DNA binding activity was analyzed by electrophoretic mobility shift assay. (E) Cancer cells were pretreated with transfected with shRNA-Calpain-10 or scramble RNA followed by stimulation with Biseugenol for 6 h. The results shown are representative of at least four independent experiments. Arrow indicates shifted DNA binding complex. The AhR DNA binding labeled probe as indicated. All experiments were repeated at least five times. (F) Interfere direct binding of AhR to the Snail promoter as demonstrated by ChIP assay. Cancer cells AGS (upper panel) or SCM-1 (lower panel) were treated with Biseugenol and then cross-linked by formaldehyde. Chromatin was immunoprecipitated with the indicated antibodies. Purified precipitates or input DNA was analyzed by PCR using primers specific for Snail (-101/−124) promoters. PCR products were subjected to gel electrophoresis and visualized by ethidium bromide staining. 10% aliquot of the pre-cleared chromatin was taken as input. All experiments were repeated at least five times.

Established as a tumor and metastasis suppressor, E-cadherin expression is frequently down-regulated or extinguished in malignancy which strongly correlates with poor prognosis. Herein, the increasing of E-cadherin is frequently considered to be a reducing the hallmark of EMT and of a blocking migratory, invasive carcinoma. However, the mechanisms underlying AhR on EMT and peritoneal dissemination remained unknown. We then examined whether AhR regulated mesenchymal (metastatic) properties controlled by Biseugenol. In addition, whether Biseugenol induced epithelial cell signature and transcriptional regulation was also determined. As shown in Figure 5D-5E, in confocal microscope image, Biseugenol significantly downregulated AhR, and phosphorylation of E-cadherin upregulated and cytokeratin-18 expression (data not shown) but not constitutive form of E-cadherin. Biseugenol-reduced EMT-related marker and transcriptional repressor Snail (Figure 6A) but not vimentin regulation was also found. Biseugenol induced cancer cells AGS and SCM-1 epithelial signature and characteristic of a mesenchymal phenotype in a dose- and time-dependent manner.

Accordingly, promoter regulation in the Snail promoter-flanking region (−89~−67) containing the *cis*-acting elements AhR DNA binding activity *in silico* was also be prospected (Figure 6B). Simultaneously, E-cadherin promoter-flanking region (−87~−68) containing the transcription repression Snail DNA binding site *in silico* was predicted in the laboratory (Figure 6C). In order to demonstrate AhR that directly regulate Snail expression, DNA binding activity of the Snail promoter was executed. As shown in Figure 6D, EMSA assay demonstrated that Biseugenol suppresses AhR binding activity on Snail promoter binding site in dose-dependent and time course. Furthermore, we investigated whether inhibition of Calpain-10 suppresses AhR cleavage and located in Snail binding site. Our results shown that treatment with calpain inhibitor or transfection with shRNA-Calpain-10 significantly was consistent with the finding that Calpain-10 induction by Biseugenol-treated regulated AhR expression result in control Snail function (Figure 6D-6E). We further to investigate the function relationship between AhR and Snail, chromatin immuno-precipitation assays were been carried out. As shown in Figure 6F, similarly, we found that Biseugenol could thwart AhR bind to the promoter of Snail and repress transcription expression. Therefore, Biseugenol is unique in its ability to induce epithelial cell signature via a AhR–Snail–E-cadherin constraint axis mechanism.

### *In vivo* biomakers of Biseugenol activity

**Figure 7:**
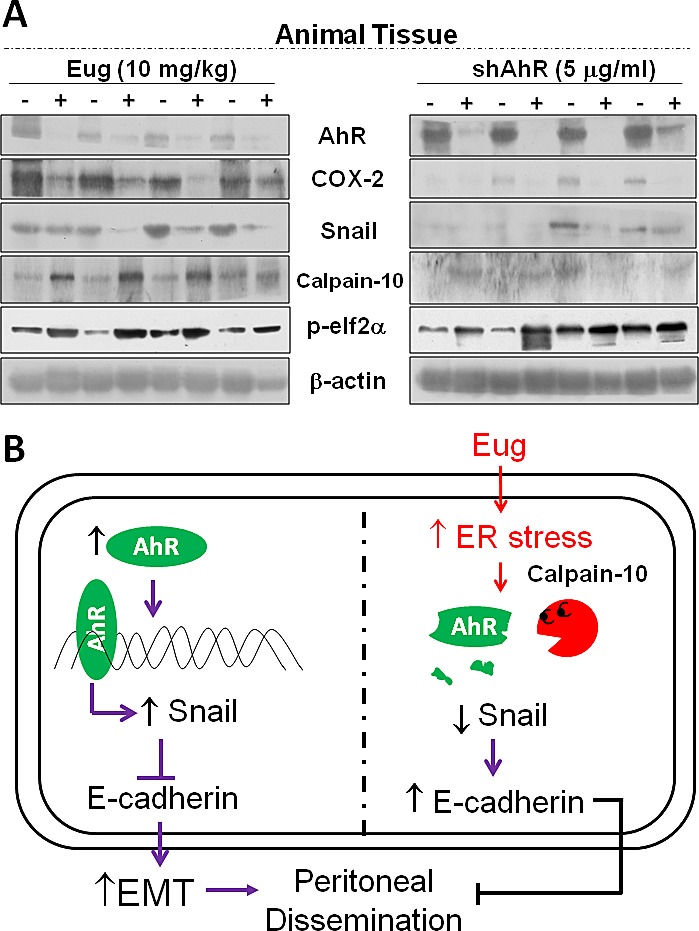
Regulation of tumor growth/dissemination-related signal molecules *in vivo* by Biseugenol or shRNA-AhR transfection (A) MKN45 cells with or without shRNA-AhR transfection were implanted by peritoneal administration to nu/nu mice in the presence or absence of Biseugenol (Eug, 10 mg/kg) treatment. Thirty days after implantation, the animals were euthanized and tumors were dissected. Targeting protein markers AhR, COX-2, Snail, p-elf2α, and Calpain-10 were evaluated by Western blotting. (B) Illustrating the working hypothesis that Biseugenol–induced ER stress was involved in the inhibition of EMT and peritoneal dissemination of gastric cancer tumors. Increased Snail signaling by AhR activation down-regulates phosphorylation of E-cadherin production. Increased Calpain-10 expression induced by Biseugenol triggers the cleavage of AhR, which subsequently inhibits Snail expression, leading to phosphorylation of E-cadherin up-regulation.

In order to verify *in vivo* hypothesis and the mechanistic findings in Biseugenol promising therapy effects, animal tissues analyses were performed. As has been shown above, the growth or peritoneal dissemination of cancer cells can be reduced by exposure with Biseugenol and by Calpain-10-induced inhibition of AhR–Snail–E-cadherin repression axis. The profile for animal tissues was similar. Our data demonstrated that suppression of AhR by shRNA-AhR or exposure Biseugenol-induced Calpain-10-activity could reduce tumor growth or peritoneal dissemination in tumor tissues (Figure 7A). The works provide a valuable biomarker that could be used in future clinical trials and to evaluate pharmacokinetics and toxicology of analogs of Biseugenol. These data aid with the hypothesis that peritoneal dissemination mediated by EMT via the AhR–Snail–E-cadherin repression axis plays a pivotal role in the progression of gastric cancer cells *in vivo*. A model of working hypothesis is presented as Figure 7B.

## DISCUSSION

In the present study, we investigated the role of Biseugenol in gastric tumor growth and peritoneal dissemination *in vivo* and *in vitro*. Our results revealed that Biseugenol-induced ER stress significantly suppressed peritoneal dissemination and organ metastasis in nude mice through specific Calpain-10 activation and AhR down-regulation. Furthermore, Biseugenol extremely impeded the development of mesenchymal feature and enhanced the expression of epithelial signature markers *in vivo* and *in vitro*, manifesting that Biseugenol targets EMT to thwart peritoneal dissemination of gastric tumors growth. In this study, we also established that Biseugenol targets AhR signaling and controls transcription repression Snail expression leading to the induction of E-cadherin phosphorylation, which accordingly represses peritoneal dissemination.

EMT refers to a biological process in which cells undergo a series of biochemical changes that induce a morphological transformation from an epithelial, polarized, adhesive state to an irregular, elongated, mesenchymal phenotype that enables migratory capacity that results in the up-regulation of transcriptional modulators such as Snail, Twist, β-catenin and Smad, Slug, C/EBPβ, NF-κB, AP-1, as well as the loss of adherence molecules, and the acquirement of new moieties essential for cell growth and progression [1;30;31]. However, although factors impart available transcription factors to cancer cells, none of them specify the concrete targeting of AhR. AhR is a member of the bHLH (basic Helix–Loop–Helix)-PAS (Per-ARNT-Sim) family of transcriptional regulators that controls the expression of a diverse set of genes. AhR, also known as the Dioxin receptor, is identified as the criminal for most toxic responses discovered after exposure to PAH (Polycyclic Aromatic Hydrocarbons), Dioxins (e.g. TCDD (2,3,7,8-tetrachlorodibenzo-p-dioxin)), and Polychlorinated Biphenyls (PCBs) [32-34]. AhR that plays an important role in the interact with signaling pathways that are mediated by NF-κB, hypoxia, estrogen receptor, other hormone receptors and retinoblastoma protein in a variety of cell types, and promotes cell proliferation [13]. AhR exists in a dormant state within the cytoplasm in association with a complex of Heat Shock Protein-90 (HSP90), HSP90 Co-chaperone p23, XAP2 and AIP, that is required for the physiological activation and interactions with various regulatory and signaling proteins, including PAS heterodimerization partners ARNT, chaperone and immunophilin-like proteins (e.g. HSP90, AIP (Aryl Hydrocarbon Receptor-Interacting Protein; p23), protein kinases and phosphatases, casein kinase-2, PKC, and coactivators [35;36]. Indeed, a constitutively active AhR expressed has been found by Andersson et al. in transgenic mice reduced the life span of the mice and induced tumors in the glandular part of the stomach, manifesting the oncogenic potential of the AhR and suggesting the receptor in regulation of cell proliferation [12]. In addition, AhR activation has been associated with an increase in cyclooxygenase-2 and chronic inflammation leading to increased cancer risk [37-41]. Increasing expression of AhR has been found in breast cancer, gastric cancer, colon adenocarcinoma and lung cancer [12;39-41]. Activation of AhR regulates the activation of the mitogen-activated protein kinase or JunD, which is critically involved in contact inhibitor, inflammation, oncogenic events and tumor progression [13]. The higher AhR activity is partially attributed to a variety of signals, including Toll-like receptor ligands, proinflammatory cytokine, and toxicant stimulates, and AhR is also able to transducer additional downstream transcription factor resulting in signaling cascade [13;16;33;35]. Environmental xenobiotics, such as polycyclic aromatic hydrocarbons, congener TCDD and PCBs, a specific activator of AhR, which also stimulated protein kinase phosphorylation and furthermore induced tumor promotion, have been shown to have strong associations with formation of cancer [13;15;16;32-36]. Halogenated biphenyls, known as PCBs, are industrial toxicants associated with the activation of AhR and downstream adverse effects, including cancer. Of interest, Biseugenol is also a biphenyl, but unlike PCBs, have electron donating hydroxyl and allyl groups in comparison to the electron withdrawing halogens of PCBs. Otherwise, there is strong evidence that it may act as a paradoxically effect in tumor suppressor. Loss-of-function studies for AhR indicate that AhR might have physiologic functions. The AhR-null phenotype included increased F-actin stress fibers, depolarized focal adhesions, and enhanced spreading and adhesion [42]. Importantly, AhR knockout mice exhibit decreased tumorigenicity and migration in a xenograft model due to downregulation of the proto-oncogene Vav3 leading to diminish in Rac1 activity in AhR−/− mouse embryonic fibroblasts. Ahr−/− dermal fibroblasts secreted higher levels of active TGFβ that increased keratinocyte migration in culture and that could account for over-activation of the TGFβ pathway and for faster wound healing in the AhR-null neo-epithelium, indicating that loss of dioxin-receptor expression accelerates wound healing *in vivo* by a mechanism involving TGFβ [43;44]. However, the mechanism underlying Ahr on EMT and cancer peritoneal dissemination remained unknown. Although previous studies suggest that AhR possesses different functions, the role of AhR in cancer cells and in regulating peritoneal dissemination remains poorly understood. In clinical, the present study shows that AhR expression is correlated with malignant progression in patient's clinicopathological data. Increased AhR staining score in the primary gastric tumor highly associated with the presence of distant metastasis. Biseugenol inhibits AhR expression that markedly diminishes tumor peritoneal dissemination and reduces tumor growth in a mouse model. Other evidence demonstrates that knockdown AhR activity causes tumor burden changes and inhibits tumor growth within 2-4 weeks. These changes are in agreement with the results of PET/CT imaging studies and nodule counts in the present study. Therefore, constitutively high levels of AhR activity may agitate cell regulation and lead to abnormal functioning in cells.

ER stress triggers UPR, a signaling pathway for adaptive response. UPR initially exerts a protective effect by lowering protein synthesis, upregulation of specific ER stress-regulated genes and inhibition of general protein translation via transient adaptation or longer-term adaptation. However, severe or prolonged ER stress results in cell death via apoptotic signaling, is triggered, which could be taken as a therapeutic stratagem in cancer therapy or used in conjunction with existing therapies. Recently evidence indicates that targeting ER stress correlation with inhibition of angiogenesis *in vivo* [2;3;5;6;21]. Our previous study has demonstrated that ER stress-induced by Honokiol consequence human gastric cancer cell apoptosis and suppresses tumorigenesis via a Calpain-II-mediated, glucose-regulated protein-94 cleavage [29]. In addition, our group also shown direct evidence that the ER were swelled and fragmented in organelles structure in Honokiol-exposure gastric cancer cells and HUVECs [5]. Furthermore, we demonstrated for the first time that Honokiol-induced STAT-3 dephosphorylation, which via calpain-II/SHP-1 interaction has a direct regulation [5]. Lastly, therapeutic inhibition of Tpl2 by Honokiol thwarts both gastric tumor growth and peritoneal dissemination by inducing ER stress and inhibiting EMT [2]. These evidences revealed that Honokiol may be beneficial in inducing ER stress and may employ its effect comparison with antiangiogenesis in gastric cancer therapy. This finding is agreement with the results reported by Banerjee A *et al.* which exhibited that UPR is required in *nu/nu* mice microvasculature for treating breast tumor with tunicamycin, further then reducing angiogenesis *in vivo* [3]. In the present work, we found that both Tunicamycin (3 and 5 μg/ml) and Eug (40 μM) induced the increase in subG0/G1 population after cell starvation for 6 hours. Both Tunicamycin (3-5 μg/ml) and Eug (40 μM) could also enhance the expressions of Grp78, p-elf2α, and DR5 and reduce the expression of AhR in gastric cancer cells. Moreover, both Tunicamycin and Eug could also enhance the ERSE activity in gastric cancer cells. However, Tunicamycin and Eug seem to be no synergic effects on these assays. Nevertheless, these results suggest that Eugenol probably have similarity with tunicamycin in inducing ER stress. Li Y and colleagues proved that downregulation of heparanase could suppress ER stress-induced invasion and migration of breast cancer cells [45]. Chiu *et al.* have also found that the combined treatment with inhibitor of histone deacetylase such as Suberoylanilide hydroxamic acid, enhances radiosensitivity and suppresses lung metastasis in breast cancer *in vitro* and *in vivo* via an ER stress-related mechanism [46]. Hence, these results indicate that targeting ER stress to induce a reversal of EMT and invasion or migration might provide a strong rationale for the development of anticancer therapeutics for the prevention of metastasis.

Calpains are calcium-dependent intracellular non-lysosomal proteases. Calpain-10 is the atypical calpains in tissues along with m- and μ-calpains, and its certain domains have been replaced or deleted. Interesting, domain IV (calmodulin-like calcium binding sites) of Calpain-10 was replaced with a divergent T domain containing no calcium-binding EF-hand structures [47]. Previous study have reported that genetic variation in the Calpain-10 gene was associated with an increased risk for type 2 diabetes mellitus in certain human population groups has been reported by Horikawa groups [48]. Hong et al have shown that Calpain-10 protein is ubiquitously expressed in tissues from insects' cells to human, in which it could potentially affect a number of tissue processes ranging from lens differentiation [47]. Moreover, it has been demonstrated *in vitro* that nonspecific calpain inhibitors ALLM (100 mM) and E-64-d (200 mM) induce alterations of glucose-induced insulin secretion in mouse islets [49]. These findings imply that Calpain-10 in pathologic conditions. However, to our knowledge, nothing is known about the protein expression or cellular function of calpain 10 or the cellular and molecular mechanisms related to ER stress in the development, progression, and metastasis of cancer still remain to be clarified. In the present work, we found that Biseugenol activates Calpain-10 activity and protein expression, but not traditional m- or μ-calpains. Transient transfection siRNA-Calpain-10 effectively reversed Biseugenol-induced ER stress, calpain activity and AhR down-regulation. In addition, *in vitro* cleavage assay in cancer cells is response to AhR cleavage fragment in a dose-dependent manner. These results provide evidence that Calpain-10 activation dampen EMT program is induced in cancer cells under ER stress induction, which can be provoked by Biseugenol-treated condition. Hence, Calpain-10 activation may be taken as another one of atypical ER stress marker.

In conclusion, our studies revealed that Biseugenol suppressed the EMT program through ER stress and the Calpain-10/AhR/Snail axis pathway. In addition, knockdown AhR activity also dramatically reduced peritoneal dissemination, which helps uncover mysteries function of cancer cells and repertory. Consistently, it has recently been shown that AhR exhibits abundant amounts, and disruption of AhR suppresses the tumor growth. Additionally, AhR may be a useful prognostic marker for gastric cancer and novel therapeutic targets for gastric cancer invasion intervention. Taken together, targeting Calpain-10 may help eliminate AhR, thereby preventing cancer progression. We suppose that future clinical trials designed to evaluate the efficacy of Calpain-10 on the prevention of peritoneal dissemination, and recurrence will be of importance.

## MATERIALS AND METHODS

Many of the methods listed here have been published previously but are repeated here for clarity.

### Synthesis of Biseugenol

Biseugenol purity was determined by Gas Chromatograph-Mass Spectrometer (GC/MS/MS). Details could be found in the Supplementary methods.

### Cell culture

Cell culture systems were used as described previously.[2;7;21;29] Human gastric cancer cell lines, AGS (moderately differentiated gastric adenocarcinoma) and MKN 45 or SCM-1 cells (poorly differentiated gastric adenocarcinoma) were supplied by the cell bank of Taipei Veterans General Hospital (Taiwan).

### Animal Xenograft tumor mouse model and Positron emission tomography–computed tomography (PET/CT)

Cell culture systems were used as described previously.[2;7;21;29] Details could be found in the Supplementary methods.

### ERSE

Cignal ERSE Reporter Assay Kit was performed as described manufacturer (QIAGEN). Dilute transfection-ready reporter, negative control, positive control formulations and relevant test nucleic acids. Post-transfection overnight, treat the transfected cells with Biseugenol and ER stress activator. Further then, assay the activities of the signaling pathways under study, utilizing the dual luciferase assay.

### Transmission electron microscopy (TEM)

TEM was performed as described previously.[5;50] Cells were treated with or without Biseugenol for 18 h, and were then harvested. Sections were stained with uranyl acetate and lead citrate in aLKBUltrostainer, and examined in a JEM 1200 EX transmission electron microscope (JEOL, Peabody, MA) at an accelerating voltage of 80 kV.

### Immunoblotting and immunoprecipitation

Immunoblotting was performed as described previously.[5;50;51] Antibodies used in the present study were listed in Table 2. Detection was performed by ECL (Amersham) and by chemiluminescence using Kodak X-Omat film.

**Table 2:** Additional antibodies used in the study

Name	Sp. (Clone number or code number)	In the work usage	Vendor
Primary antibody			
IRE1α	sc-20790 rabbit polyclonal IgG	WB	Santa Cruz Biotechnology
GADD153	sc-7351 mouse monoclonal IgG_1_	WB	Santa Cruz Biotechnology
α-tubulin	sc-8035 mouse monoclonal IgM	WB	Santa Cruz Biotechnology
Calpain I	sc-7531 goat polyclonal IgG	WB	Santa Cruz Biotechnology
Calpain II	sc-7533 goat polyclonal IgG	WB	Santa Cruz Biotechnology
Calpain X	sc-48454 goat polyclonal IgG	WB	Santa Cruz Biotechnology
p-PERK(Thr981)	sc-32577-R rabbit polyclonal antibody	WB	Santa Cruz Biotechnology
p-eIF2α(Ser51)	9721 polyclonal antibodies	WB	Cell Signaling
Aryl hydrocarbon receptor	sc-5579 rabbit polyclonal IgG	WB,IP,IHC(P)	Santa Cruz Biotechnology
Arnt1	Sc-5580 rabbit polyclonal IgG	WB	Santa Cruz Biotechnology
p-E-cadherin(Ser838+Ser840)	ab76319 monoclonal antibody	WB,IF,IHC(P)	Abcam
E-cadherin	sc-7870 rabbit polyclonal antibody	WB,IHC(P)	Santa Cruz Biotechnology
E-cadherin	3195 monoclonal antibody	WB	Cell Signaling
Snail	sc-28199 rabbit polyclonal IgG	WB,IHC(P)	Santa Cruz Biotechnology
Cytokeritin18	sc-32329 mouse monoclonal IgG 1	WB,IHC(P)	Santa Cruz Biotechnology
P-cadherin	C13F9 monoclonal antibody	WB	Cell Signaling
Caspase 3	sc-1226 goat polyclonal IgG	IHC(P)	Santa Cruz Biotechnology
Secondary antibody			
Fluorescein-Labeled Antibody To Rabbit IgG (H+L)	02-15-16	IF	Kirkegaard&Perry Laboratories, Inc
Doenkey anti-goat HRP	sc-2020	WB	Kirkegaard&Perry Laboratories, Inc
Peroxidase-conjugated AffiniPure	115-035-003	WB	Jackson ImmunoResearch Laboratories, Inc.
Peroxidase-conjugated AffiniPure goat anti-rabbit IgG (H+L)	111-035-003	WB	Jackson ImmunoResearch Laboratories, Inc.
Rhodamine-Labeled Affinity Purified Antibody To Mouse IgG (H+L)	03-18-06	IF	Kirkegaard&Perry Laboratories, Inc
Rhodamine-Labeled Affinity Purified Antibody To Rabbit IgG (H+L)	03-15-06	IF	Kirkegaard&Perry Laboratories, Inc
Fluorescein-Labeled Antibody To Mouse IgG (H+L)	172-1806	IF	Kirkegaard&Perry Laboratories, Inc

Abbreviations: WB: Western blot; IF: immunofluorescenece; IHC(P):immunohistochemistry(paraffin

### Calpain activity assays

Calpain activity assay was performed as described previously [5;50]. Assay was done using fluorogenic peptide substrate (Suc-Leu-Tyr- AMC) analyzed on a fluorescence plate reading system (HTS-7000 Plus Series BioAssay, Perkin Elmer) with filter settings of 380 ± 20 nm for excitation and 460 ± 20 nm for emission.

### Electrophoretic mobility shift assay (EMSA)

The electrophoretic mobility shift assay was performed as described previously [5;50;52]. The oligonucleotide with the AhR consensus binding sequence used were AhR/Snail-62~-85 (5′ GGGGCGTGGCAGATAAGGCCCCG3′). DNA–protein complexes were resolved on 6% nondenaturing polyacrylamide gels and visualized by exposure to autoradiographic films.

### Immunofluorescence and laser scanning confocal microscopy

Cells were prepared and the immunofluorescence was determined by laser scanning confocal microscopy (LSCM, TCS SL, Leica, Wetzlar, Germany) as previously described.[5;50;52] Images were background-subtracted and merged using the Confocal Assistant MetaMorph software program, and processed with Adobe Photoshop software.[51;52]

### *In vitro* cleavage assay

*In vitro* proteolysis of AhR by Calpain-10 was analyzed by a modified procedure as described previously.[52] Purified protein was incubated with recombinant Calpain-10 (Calbiochem) in a reaction buffer at 30°C for 4 h. The reaction mixtures were then loaded on a 10% SDS-PAGE gel. The cleavage of AhR by Calpain-10 was analyzed by Coomassie blue staining of the gel and immunoblotting.

### Chromatin immunoprecipitation assay (ChIP)

The ChIP assay protocol was modified from the description by Latasa et al.[53] A fragment of the (220 b.p.) Snail promoter containing putative AhR-binding sites. The primers used, -101~-124, 5′ C GGC GGA GAC GAG CCT CCG ATT G 3′; +75~+96, 5′ GGA AAG AGC GCG GCA TAG TGG 3′.

### Statistical analyses

The values were presented as mean ± SEM. Analysis of variance, followed by Fisher's least significant difference test, was performed for all data. Statistical significance was set at *p*<0.05.

## SUPPLEMENTARY MATERIAL FIGURES


