# Supplementary material for: The novel Aryl hydrocarbon receptor inhibitor biseugenol inhibits gastric tumor growth and peritoneal dissemination
Source: Oncotarget. 2014 Aug 4;5(17):7788–804. doi: 10.18632/oncotarget.2307 (PMC4202161; doi:10.18632/oncotarget.2307)
Supplement: Supplementary file 1 [file oncotarget-05-7788-s001.pdf]

The novel Aryl hydrocarbon receptor inhibitor biseugenol inhibits gastric tumor growth and peritoneal dissemination

Supplementary Material

Synthesis of Biseugenol

NMR spectra were recorded in deuterated chloroform ($CDCl_3$) with a Varian INOVA 400 MHz instrument, calibrated using residual undertreated chloroform (^{1}H: δ = 7.24 ppm) as internal standard. The following abbreviations, or a combination thereof, are used to explain the multiplicities: s = singlet, d = doublet, t = triplet. High resolution mass spectrometry (HRMS) analysis was performed with a Thermo Scientific LTQ FT Ultra Hybrid mass spectrometer set on positive ionization. Biseugenol was synthesized by dimerization of Biseugenol according to the procedure described by de Farias.[1] Clove oil from Matheson Coleman & Bell (Gardena, CA) was used as the source for Biseugenol. Clove oil (1.0 g, 5.5 mmol, 90% Biseugenol) was dissolved in acetone/H_2O 2:1 (30 mL), NH_4OH (aq, 18 mL, 29%) was added and the mixture was stirred at room temperature for 10 minutes. A saturated aqueous solution of $K_3Fe(CN)_6$ (2.0 g, 6.1 mmol) was added drop wise over a period of 4 hours, followed by another addition of NH_4OH (aq, 18 mL, 29%). The mixture was stirred at room temperature for an additional 18 hours and then neutralized by drop wise addition of HCl (aq, 10%). A precipitate was formed, which was filtered off, dissolved in acetone, dried over Na_2SO_4, filtered, and concentrated under reduced pressure. The crude product was purified by column chromatography on silica gel (ethyl acetate/hexanes 1:3), which afforded the product as a white powder (0.67 g, 75%). The obtained ^{1}H NMR was according to previous work.

Animal Xenograft tumor mouse model and Positron emission tomography–computed tomography (PET/CT)

Cell culture systems were used as described previously [2;5;7].The tumor was successfully inoculated, then the mice were injected intra-peritoneal with Biseugenol inhibitor (10 mg/kg, twice per week). Images were clarified by experienced nuclear medicine physicians with all available clinical information and correlative conventional imaging was used for anatomic guidance. Consecutive surveillance PET/CT images from 7, 14, 21, and 28 days after execution with Biseugenol were treated to detect peritoneal dissemination. The cells (5×10^6) were transfected with siRNA using Lipofectine. The MKN45 siRNA-AhR and MKN45 Scramble cells were transected for 24 h and then each of the mice received one of the cell types via intraperitoneal injection to the abdominal cavity for 30 days. Quantification of the vessels was estimated by analyzing five randomly chosen high-power fields. The mice were sacrificed under anesthesia (pentobarbital) and examined macroscopically for the presence of peritoneal metastasis. The tumors were excised, cut into blocks, fixed in 10% formalin, and embedded in paraffin blocks or snap-frozen in liquid nitrogen.

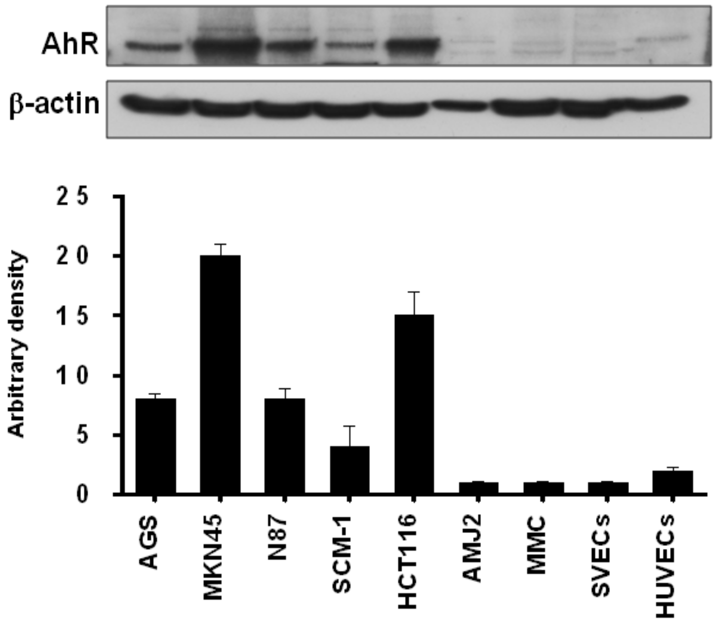

Supplementary Fig. 1: AhR expression in different human stomach cancer epithelial cell lines. Protein level of AhR protein in cultured human stomach cancer epithelial cell lines (AGS, MKN45, N-87, SCM-1), human colon cancer epithelial cell line (HCT116) and normal cells (AMJ2, MMC, SVECs, HUVECs). Beta-actin was used as a loading control. Quantification of relative protein levels on three different western blots is shown below the blots. The results were from a representative of at least three repeated experiments.

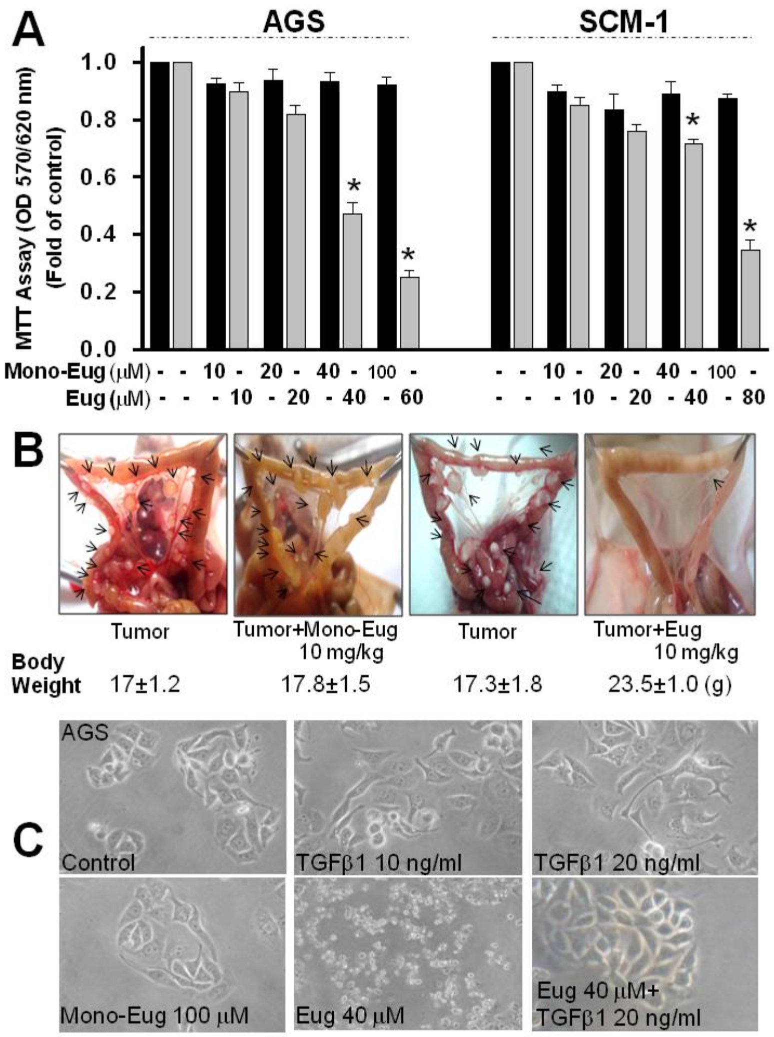

Supplementary Fig. 2: Biseugenol , but not Monoeugenol, reduces cell proliferation and thwarts peritoneal dissemination. (A) AGS or SCM-1 cells were treated with Biseugenol (Eug) or Monoeugenol (Mono-Eug) alone for 24 h. Cell viability was measured. The results shown are representative of at least three independent experiments. (B) Mice were then injected intraperitoneally with Biseugenol or Monoeugenol (10 mg/kg/twice per week, n=4). Twenty-eight days after Biseugenol or Monoeugenol treatment, mice were sacrificed for macroscopic examination of the distribution of disseminated metastasis. Body weights as indicated. (C) Biseugenol inhibits TGFβ-induced EMT. AGS cells were pretreated with Biseugenol or Monoeugenol and further then stimulated with 10-20 ng/ml TGFβ for up to 24 h. Phase contrast micrographs were taken with Olympus CKX41 40 phase contrast microscope. The results were from a representative of at least three repeated experiments.

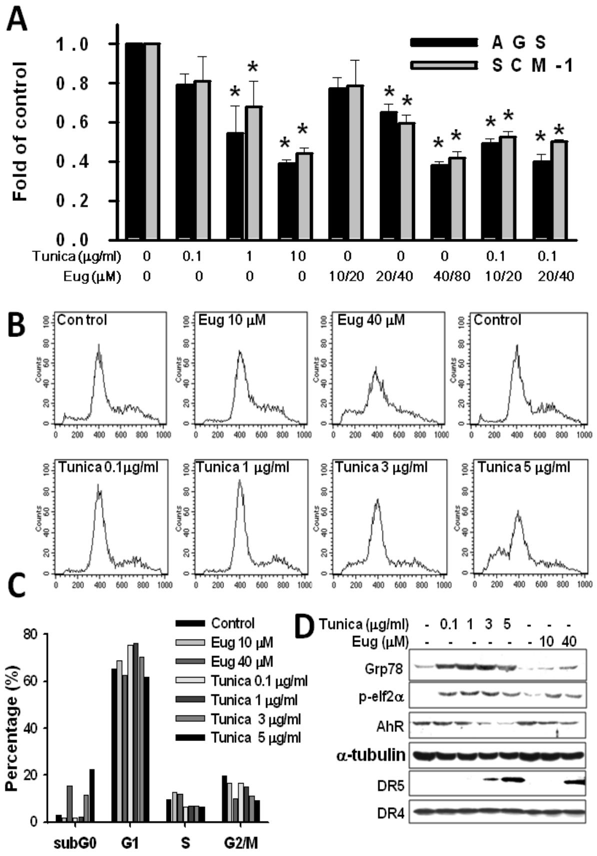

Supplementary Fig. 3: Biseugenol or Tunicamycin induces apoptosis and ER stress in gastric cancer cells. (A) AGS or SCM-1 cells were treated with Biseugenol (Eug) or Monoeugenol (Mono-Eug) alone for 24 h. Cell viability was measured. The results shown are representative of at least three independent experiments. AGS or SCM-1 cells were cultured with tunicamycin (0. 1-10 μg/mL) or Biseugenol (10- 80 μM) for 24 hours. Then the cells undergoing apoptosis was studied using MTT assay or (B) flow cytometry detection. (C) Biseugenol or Tunicamycin induced cell death for 24 hours. After starvation 6 hours, cell cycle was then studied using PI staining by flow cytometry. Percentages indicate cells in sub-G0 phase, G1 phase, and G2/M phase for 0.1-5μg/mL tunicamycin and Biseugenol 10-40 μM. The data represent quantification of cell cycle. (D) Biseugenol or Tunicamycin induces AGS cells apoptosis for 24 hours. Cells were cultured with as indicated then whole-cell lysates were subjecting to Western blotting using ER stress marker anti–Grp78, p-elf2α, and α-tubulin antibodies was used as the loading control. Death receptor DR4 or DR5 and AhR was measured. All the results shown are representative of at least four three experiments.
